# Using modified mRNA for cardiomyocyte proliferation and cardiac genetic disease modelling and treatment

**DOI:** 10.1042/BST20243001

**Published:** 2025-09-04

**Authors:** Christopher A.P. Batho, James E. Hudson, Catherine H. Wilson

**Affiliations:** 1 Department of Pharmacology, University of Cambridge, Cambridge, CB2 1PD, U.K; 2 QIMR Berghofer Medical Research Institute, Herston, QLD, 4006, Australia

**Keywords:** cardiac hypertrophy, cardiomyocytes, cell proliferation, genome editing, MRNA, regeneration

## Abstract

Heart failure (HF) is a leading cause of death worldwide and the associated mortality and socioeconomic burden is predicted to worsen. Current therapies for HF focus on managing the causes and symptoms; however, these current treatment options are unable to reverse heart muscle degeneration, with heart transplantation the only cure. The ability to re-muscularise the heart represents a significant unmet clinical need. Although numerous biological pathways driving re-muscularisation have been identified, delivery of therapeutic factors is challenging. Modified mRNA (modRNA) is synthetic mRNA with greater gene packaging capacity, low immunogenic response and allows transient but robust protein expression. In this mini-review, we highlight the emerging discoveries surrounding the application of modRNA in the cardiovascular field. Specifically, we focus on different examples illustrating how modRNA delivery post-myocardial infarction can drive cardiomyocyte proliferation and achieve cardiac regeneration. In addition, we demonstrate how modRNA is being used for protein replacement and Cas delivery for both modelling and therapeutic studies focussed on genetic cardiac diseases. For these applications, in particular Cas delivery, the transient nature of modRNA overexpression is a beneficial property with reduced side effects compared with other modalities. Finally, we preview some of the roadblocks limiting the clinical translation of modRNA and avenues being explored to overcome these. In summary, the flexibility of modRNA combined with its improved safety profile provides a gene overexpression tool capable of integration into all steps of the preclinical and clinical therapeutic pipeline enabling the discovery of improved treatments for HF.

## Introduction

Heart failure (HF) is characterised by a reduced capacity of the heart to fill and/or pump blood [1] and is a leading cause of death worldwide, affecting an estimated 64 million people [2]. Patient numbers are projected to increase in the coming years, placing a huge burden on the healthcare system with treatment costs predicted to be $70 billion by 2030 in the US alone [3]. Aside from the financial impact, HF significantly diminishes the quality of life of those afflicted, making it clear that improved treatment options are required. Currently, first-line treatment for HF patients is pharmacotherapy, which focuses on symptom control by reducing blood pressure (e.g. ACE inhibitors) and blood volume (e.g. loop diuretics). However, these interventions do not address the underlying cause of disease and ultimately do not prevent disease progression [4]. As our understanding of the molecular mechanisms governing HF has improved vastly in recent decades, research has provided promising novel targets for therapeutic intervention. To manipulate these novel targets, multiple gene therapy methods have been employed including plasmids, proteins and viruses (lenti-, adeno- and adeno-associated). However, these approaches can cause random genome integration, ineffective translation due to pre-existing neutralising antibodies and/or uncontrolled gene expression limiting their application [5].

A therapeutic modality that overcomes these drawbacks is modified mRNA (modRNA). The use of modRNA has been recently highlighted due to its involvement in the swift development of vaccines to combat the COVID-19 pandemic [6,7]. ModRNA translation is transient, allowing for short-term, robust expression of one or multiple proteins. This synthetic mRNA has an enhanced gene packaging capacity and induces minimal immunogenic response [8,9]. Aside from viral vaccine development, modRNAs have been designed to treat varying pathologies including metabolic disorders, cystic fibrosis and multiple cancers [10]. Therapeutic development has been extended to cardiovascular diseases with VEGF-A modRNA therapy recently completing a phase 2a clinical trial [11]. This review will focus on the emerging field of modRNA and its use in therapeutics for driving cardiomyocyte (CM) proliferation as well as treating and modelling genetic diseases.

## Viral vs non-viral gene therapy

Traditional use of pharmacotherapy to treat HF has seen drug cocktails developed to regulate fluid retention, reduce cholesterol levels and manage contraction, which has halved mortality in moderate HF patients [12]. These approaches have continued to evolve with the use of SGLT2 inhibitors [13] and cardiac myosin activators [14], further improving HF treatment strategies. However, pharmacological intervention does not correct the underlying cause of the disease and is often accompanied by side effects. With increasing knowledge of the molecular drivers of HF, the use of gene therapy has revolutionised HF therapeutic options (reviewed in [15]).

Gene therapy involves the delivery of genetic material (Table 1) to cells resulting in gene repression, overexpression or editing depending on the approach taken and pathology in question. The use of DNA-based vectors has dominated the gene therapy field and can be distributed using non-viral or viral vectors. Using a non-viral vector, plasmid DNA has been utilised due to its expression period of one to four weeks and low immunogenicity, which has seen numerous clinical trials undertaken (reviewed in [16]). However, plasmids exhibit low transfection efficiency [17], whilst a lack of GMP-grade manufacturing facilities exists [18], restricting their clinical application.

**Table 1:** Advantages and disadvantages of different gene therapy delivery vectors

	Plasmid	Retrovirus	Lentivirus	Adenovirus	AAV	modRNA
Advantages	Enhanced gene packaging capacity Expression duration (1–4 weeks) Simple to construct and propagate Low immunogenicity	Intermediate expression duration (months) Low immunogenicity Very low pre-existing immunity Stable genome integration	Long-term expression duration (months to years) High transfection efficiency Stable genome integration	Quickest transgene expression amongst viral vectors New-generation viruses have enhanced gene packaging capacity (~30 kb) No genome integration	Long-term expression duration (months to years) Low immunogenicity Wide-cell tropism Drug-inducible systems providing temporal control	Enhanced gene packaging capacity Low immunogenicity Rapid, transient expression No genome integration Short synthesis time
Disadvantages	Low transfection efficiency Presence of antibiotic resistance genes Upscaled, GMP-grade manufacture restricted	Unable to transfect non-dividing cells Risk of insertional mutagenesis Limited transgene capacity (~8 kb)	Longer time until peak expression Risk of insertional mutagenesis Limited transgene capacity (~8 kb)	Intermediate expression duration (weeks to months) Highly immunogenic Prevalence of neutralising antibodies in population	Potential for insertional mutagenesis Small transgene capacity (5 kb) High prevalence of neutralising antibodies in population	Short-lasting expression prevents certain clinical applications Cell-type specific expression requires further optimisation Delivery mechanisms require optimisation

The development of viral vectors has provided a way to circumvent transfection issues whilst providing longer expression of the transgene. Retroviruses undergo stable genome integration which enables transgene expression for many months [19]. However, they are unable to transfect non-dividing cells [20], limiting their use with CMs. Lentiviruses are a complex retrovirus which can transduce postmitotic and quiescent cells [21] and provide expression for many months [22]. However, like its family members, they have an increased risk of insertional mutagenesis [23] affecting their safety profile. Adenoviruses have been the most used viral vector for clinical trials thus far and rarely undergo genome integration, demonstrating rapid expression of transgenes up to 35 kb in size [24]. Whilst being popular in the preclinical and clinical pipeline, adenoviruses are highly immunogenic and offer expression for only weeks to months (reviewed in [25]). The viral vector with the most FDA-approved therapies [26] is adeno-associated viruses (AAVs) which have been used prominently in preclinical cardiac studies (reviewed in [27]). AAVs demonstrate low immunogenicity and transgene expression lasting up to 15 years [28], making them an attractive vector for genetic disorders. However, AAVs possess a small gene packaging capacity and neutralising antibodies are prevalent in the community (reviewed in [29]). Additionally, the prolonged expression has been shown to be detrimental in a cardiac regeneration study with arrhythmias causing death in a porcine model [30]. To overcome these drawbacks, recent work has seen the development of drug-inducible AAV systems whereby administration of splice-modulating drugs enables transgene expression providing temporal control [31] and has already been applied to the cardiac regeneration field [32].

Although DNA delivery using viral vectors has been the standard for cardiac gene therapy, the issues around insertional mutagenesis, pre-existence of neutralising antibodies and the relatively small transgene capacity still ultimately restrict their application. Compared with DNA expression strategies, modRNAs are unable to integrate into the genome and have no gene packaging capacity [33]. Further improving the safety profile, the introduction of modified caps and nucleosides diminishes the immunogenic response [9], and they are degraded within a short timeframe compared with DNA, making them an increasingly appealing gene therapy modality.

## Modified mRNA

The first published use of exogenous mRNA for gene overexpression was recorded in 1989 [34] and was soon followed with *in vivo* delivery of chloramphenicol acetyltransferase mRNA to skeletal muscle in 1990 [35]. Following these and other proof of principle experiments, the majority of research using exogenous mRNA for therapeutic approaches was applied to vaccine development for viral infections [36] and cancer immunotherapy [37]. However, the clinical application of exogenous mRNA was limited by its instability and immunogenic properties. When a cell is subjected to foreign DNA or RNA, an inflammatory response arises due to the innate immune system whereby retinoic acid-inducible gene-I-like receptors [38] and toll-like receptors [39] recognise the foreign nucleic acid driving interferon [40] and tumour necrosis factor (TNF) production [41]. For instance, when monocyte-derived dendritic cells were transfected with different types of RNA, unmodified mRNA induced similar levels of TNF production as bacterial RNA and mammalian mitochondrial RNA [8]. This activation potential displayed an inverse correlation with the degree of nucleoside modification, with bacterial and mitochondrial RNA exhibiting few modifications. When exogenous mRNA was synthesised with modified nucleosides, the inflammatory response was suppressed in a proportional manner to the number of modified nucleosides present. It was this seminal work by Katalin Karikó and Drew Weissman that enabled the development of the first FDA-approved mRNA-based biologic vaccine in 2020 to combat COVID-19 [42] and ultimately earned them the 2023 Nobel Prize in Physiology or Medicine. Reducing the immunogenicity elicited by mRNA allowed the use of exogenous modRNA to extend into other therapeutic strategies including neutralising antibodies [43–46], genome editing [47–49] and protein replacement [50–53].

**Figure 1:**
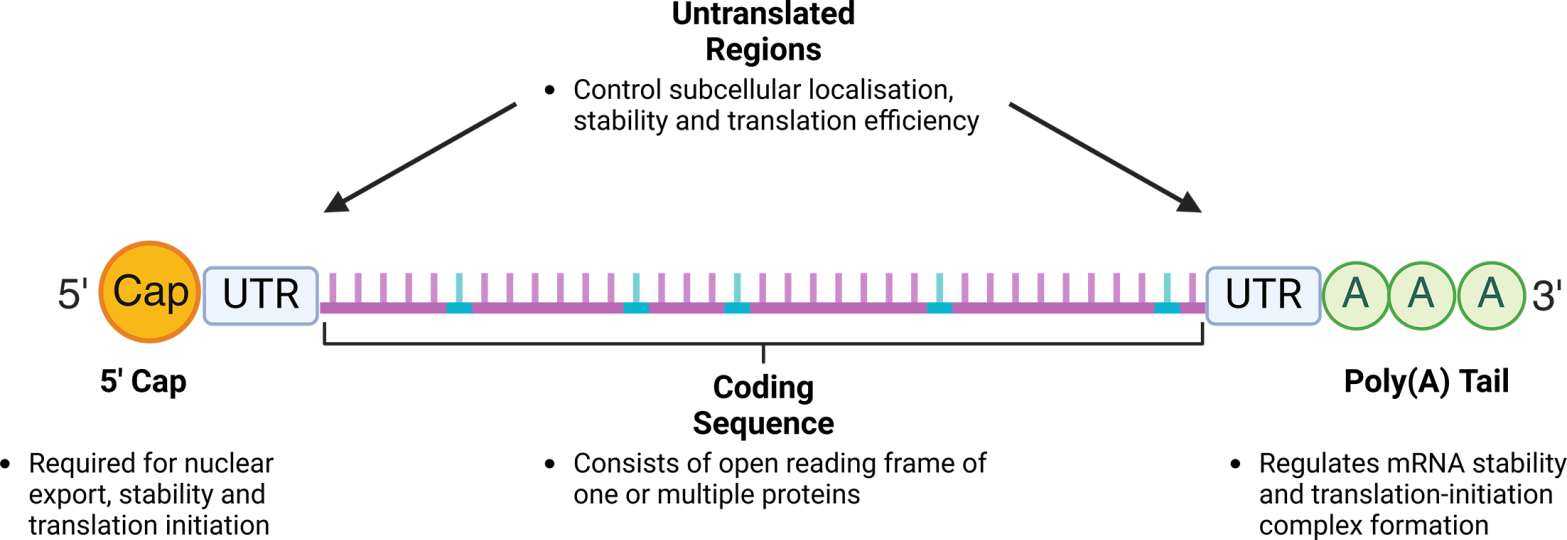
ModRNA structural components. ModRNA shares the classical features of mRNA; however, its design is crucial to optimal protein expression. The 5′ cap can be added co- or post-transcriptionally with Cap 1 analogues (e.g. CleanCap) minimising immune response. As the UTRs help control mRNA stability, the α- and β-globin genes have been traditionally used in modRNA design. Through UTR optimisation, it has been uncovered that secondary structure in the 5' and 3' UTR has negative and positive effects on translation, respectively. For the coding sequence, development of mRNA design algorithms has enabled the optimisation of structural stability and codon usage to produce the optimally translated mRNA. Furthermore, incorporation of modified nucleosides (cyan) reduces immunogenicity and enhances translation. Finally, the poly(A) tail can be introduced during or after IVT with a poly(A) of 80 adenosines producing protein levels comparable with the standard 120 adenosines. Figure created with Biorender.com.

The structural composition of modRNA shares the classical features of any mature mRNA (Figure 1). The 5′ 7-methyl-guanosine cap is essential for mRNA functionality by regulating nuclear export [54], stability [55] and translation initiation [56]. For modRNA, the cap can be introduced co-transcriptionally or post-transcriptionally. With the former, the polymerase incorporates a cap analogue during *in vitro* transcription (IVT) leading to capping efficiencies between 50-95% depending on analogue type. Early cap analogue iterations, such as anti-reverse cap analogue, produce a cap 0 structure which leads to immune activity without an extra methylation reaction [57]. The development of CleanCap, which provides a cap 1 structure containing this methylation group, minimises immune activation, increases capping efficiency and yield [58]. Conversely, capping post-IVT can be achieved using Vaccinia virus which results in 100% capping efficiency [59] but at the expense of further handling and purification.

Flanking the coding sequence are the untranslated regions (UTRs) which regulate post-transcriptional processes including subcellular localisation [60], mRNA stability [61] and translation efficiency [62]. As the 5′ UTR is crucial for translation initiation, multiple studies focused on 5′ UTR design have highlighted the importance of sequences lacking multiple start and stop codons [63] as well as avoiding secondary structure formation to enable effective mRNA-ribosome binding [64]. Conversely, secondary structures in the 3′ UTR correlate with elevated protein expression [64]. For 3′ UTR design, the traditional approach has been the use of abundantly expressed genes such as α- and β-globin to provide a stable transcript [65]. However, recent work has shown a modRNA with a four-base 5′ UTR and no 3′ UTR produced functionally comparable protein with a β-globin 3′ UTR illustrating further optimisation is possible [66].

The poly(A) tail is required for mRNA stability [67] and formation of a closed-loop structure in the translation-initiation complex [68]. Similar to the 5′ cap, the poly (A) tail can be introduced co-transcriptionally based on the DNA template or post-IVT using a poly(A) polymerase [69]. The consensus has seen modRNA poly(A) length to be 100–120 adenosines in length to enable increased mRNA stability and translational efficiency via efficient poly(A) protein binding [70,71]. However, recent work has illustrated that a modRNA with 80 adenosines produces protein at levels equivalent to 120 adenosines [72], indicating that binding of at least two poly(A) binding proteins is sufficient to protect from deadenylation [73].

The component that enables the widespread application of this modality is the coding sequence which consists of the open reading frame (ORF) of one or multiple proteins depending on the gene therapy application. Aside from the ability to incorporate any ORF, there are several modifications to the coding sequence which have major implications on mRNA stability and translation. This includes the choice of modified nucleosides for any of the four bases which can diminish the cellular immune response [8,74] and enhance protein expression [9,72]. Additionally, recent work has highlighted the importance that codon optimisation and mRNA structure play in translation and stability, respectively. Development of multiple online tools [75–77] has streamlined this process, enabling the design of optimally translated mRNA to achieve elevated protein expression. These wide-ranging considerations in the design of modRNA make it an extremely versatile system where its application has been observed for both cardiac regenerative and genetic disease research.

## Modified mRNA for CM proliferation

With cardiovascular diseases the leading cause of death worldwide [78], modRNA has begun to be utilised for therapeutic development in this space. After numerous studies focussed on delivery optimisation (reviewed in [79]), myocardial infarction (MI) has been the primary disease model for cardiovascular modRNA therapeutic development. After an MI, several pathological processes occur, including CM death, immune cell infiltration and myofibroblast activation compounded by insufficient neovascularisation (reviewed in [80]). Multiple studies using modRNA have demonstrated its ability to address these perturbations through reducing CM apoptosis [81,82], inflammation [83] and cardiac fibrosis [84–86], whilst also promoting angiogenesis [87]. However, the most advanced therapeutic target for cardiovascular modRNA medicine is VEGFA which drives neovascularisation [11,88–91] and improves CM engraftment [92]. Whilst replacement of lost CMs through fibroblast reprogramming has been demonstrated using modRNA [87,93], this review will focus on modRNA therapeutics targeting CM proliferation.

**Figure 2:**
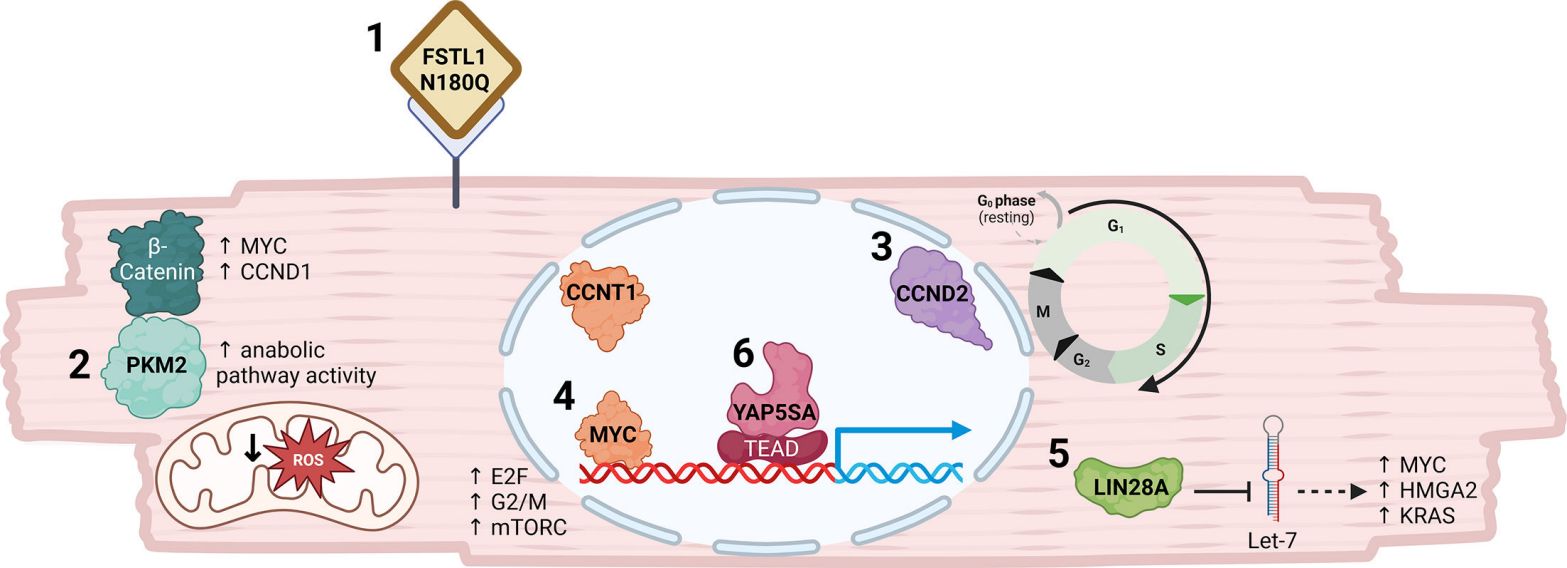
Schematic of cardiomyocyte illustrating effects of modRNAs that drive proliferation. ModRNAs used to drive cardiomyocyte proliferation target different biological mechanisms to achieve cardiac regeneration [1]. In cardiomyocytes, FSTL1 N180Q binds an unknown receptor that ultimately results in increased proliferation[2]. PKM2 overexpression drives cardiomyocyte proliferation by binding β-catenin and increasing anabolic metabolism resulting in up-regulated cell cycle gene (MYC and CCND1) expression and decreased reactive oxygen species (ROS) [3]. Overexpression of CCND2 overcomes cell cycle arrest by driving the G1/S transition allowing DNA synthesis and cell cycle progression [4]. MYC and CCNT1 causes up-regulation of protein expression of genes related to mTORC1, E2F targets and the G2/M checkpoint [5]. LIN28A binds and inhibits Let-7 microRNAs enabling increased expression of MYC, HMGA2 and KRAS which are required for cardiomyocyte proliferation [6]. YAP5SA binds to its transcriptional co-activator TEAD which binds to promoters enabling gene expression. Figure created with Biorender.com.

In the first week of postnatal life, CM maturation occurs, coinciding with reduced capacity for CM proliferation, resulting in poor regenerative potential within adult mammals. This short regenerative window was first demonstrated in mice [94] and has since been observed in rats [95], rabbits [96], sheep [97], pigs [98,99] and anecdotally in newborn humans [100,101]. When regeneration is possible, pre-existing CMs undergo proliferation to replace the damaged tissue [94], making strategies targeting CM cell cycle re-entry an attractive therapeutic approach. As uncontrolled CM cell cycle re-entry can lead to arrhythmia and death [30], the transient, pulse-like nature of modRNA makes it ideal for cardiac regenerative gene therapy. Currently, there are six published articles or preprints using modRNA to drive CM proliferation which we will preview here (Table 2, Figure 2). These studies highlight the potential of using modRNA as a vector for gene therapy to drive CM proliferation both *in vitro* and *in vivo*. In addition to achieving improved cardiac function through increased CM proliferation, the transient nature of modRNA was demonstrated with no prolonged CM proliferation (four weeks post-MI) quantified in several of the studies reviewed here. This finding is important as it indicates that modRNA therapies will likely prevent persistent CM dedifferentiation which, if uncontrolled, has led to arrhythmogenesis and/or decreased cardiac output in zebrafish [118], mice [119–121] and pigs [30].

**Table 2:** Published articles or preprints using modRNA for cardiomyocyte proliferation for cardiac regeneration

Target/s (Gene)	Known target function	*In vitro* effects	*In vivo* delivery and injection method	*In vivo* effects
Follistatin-like 1 protein, N180Q mutant [FSTL1] [102]	Secreted glycoprotein; bacterially vs. mammalian-produced protein results in cardioprotective vs. regenerative role, respectively [103].	Overexpression of 3 glycosylation-null FSTL1 mutants increased neonatal rat CM (NRCM) proliferation evidenced by increased BrdU+, Ki67+, pHH3+ and AURKB+ CMs.	100 µg/modRNA in sucrose citrate buffer was injected intramyocardially during mouse LAD ligation surgery.	Only the FSTL1 N180Q mutant modRNA significantly elevated CM proliferation. FSTL1 N180Q improved ejection fraction, reduced scar size and increased heart weight but not through hypertrophy.
Pyruvate kinase isozyme 2 [PKM2] [104]	Glycolytic enzyme that catalyses final step of glycolysis; up-regulated after MI and interacts [105] with known pro-proliferative gene, β-catenin [106].	CM-specific PKM2 modRNA significantly increased NRCM proliferation indicated by elevated BrdU+, Ki67+, pHH3+ and AURKB+ CMs. Effects achieved through β-catenin binding and elevated anabolic pathway activity resulting in up-regulated CM cell cycle gene expression and reduced oxidative stress, respectively.	100 or 150 µg/modRNA in sucrose citrate buffer was injected intramyocardially during mouse LAD ligation surgery or 15 days post-MI (chronic).	CM-specific PKM2 modRNA injected immediately post-MI increased CM proliferation and improved cardiac function, reduced scar size and improved survival. In a chronic model, PKM2 modRNA delivered 15 days post-MI also led to increased CM mitosis and ejection fraction.
Cyclin D2 [CCND2] [107]	Cyclin D2 (CCND2) regulates the G1-S phase transition of the cell cycle [108] and has been shown to increase CM proliferation [109] and improve function post-MI [110].	CM-specific CCND2 overexpression for 2 days in (hPSC-CMs) resulted in increased CM proliferation evidenced by increased Ki67+, BrdU+, pHH3+ and AURKB+ CMs and total CM number.	150 µg/modRNA (mouse) or 7.5 mg/modRNA (pig) in sucrose citrate buffer was injected intramyocardially during LAD ligation surgery.	CM-specific CCND2 modRNA increased both CM proliferation and cardiac function accompanied by smaller infarcts. In pigs, CCND2 modRNA promoted a controlled increase in CM proliferation which reduced scar size, ultimately resulting in improved cardiac function 4 weeks post-injury.
Myc and cyclin T1 [MYC and CCNT1] [111]	Myc is a pleiotropic proto-oncogene that co-ordinates a variety of transcriptional programmes [112] whilst CCNT1 is required for RNA polymerase II activity [113].	Myc-CCNT1 modRNA induced CM proliferation in hPSC-CMs and increased E2F targets and G2-M checkpoint signatures at the protein level.	100 µg/modRNA in Lipofectamine RNAiMAX/PBS was injected intramyocardially during mouse LAD ligation surgery.	Myc-CCNT1 modRNA delivered post-MI improved ejection fraction and increased muscle area due to elevated CM proliferation indicative of regeneration.
Protein lin-28 homolog A[LIN28A][114]	LIN28A is an RNA-binding protein that regulates metabolism and promotes proliferation through Let-7 pre-microRNA suppression [115]	CM-specific LIN28A modRNA increased pHH3+ CMs and elevated expression of MYC, HMGA2 and KRAS – which are Let-7 regulated. Silencing expression of these genes ablated pro-proliferative effects of LIN28A.	Dosage not specified. modRNA injected intramyocardially during mouse LAD ligation surgery.	CM-specific LIN28A modRNA treatment reduced scar size, improved cardiac function and survival and was accompanied by elevated pHH3+ CMs. LIN28A overexpression decreased expression of Let-7 pre-microRNA.
Yes-associated protein, 5SA (active) mutant [YAP5SA] [116]	YAP is a regulator of the Hippo pathway with over-expression driving cardiac regeneration [117].	CM-specific YAP5SA modRNA drove CM proliferation as seen by increased Ki67+, pHH3+ and AURKB+ CMs. Overexpression drove a less mature phenotype indicated by reduced expression of contractile and calcium handling genes.	150 µg/modRNA in sucrose citrate buffer was injected intramyocardially during mouse LAD ligation surgery.	CM-specific YAP5SA modRNA resulted in improved cardiac function post-MI and reduced infarct size. This was achieved through increased Ki67+ and pHH3+ CMs and a decrease in CM size indicating CM proliferation.

These findings are encouraging and demonstrate how the transient expression of modRNA is suitable for cardiac regenerative purposes. Whilst encouraging, the timing of therapeutic intervention is crucial and is a major difference between preclinical and clinical models. To this point, most *in vivo* regenerative studies in mice have seen therapeutic administration occur simultaneously with ligation of the left anterior descending (LAD) coronary artery and no subsequent reperfusion. In contrast, most patients suffering a MI receive immediate heart reperfusion to restore blood flow, resulting in recovery times of at least 8 weeks [122], during which significant ventricular remodelling and fibrosis occurs [123]. The consequences of these perturbations on modRNA efficacy have not been fully investigated to see if CM proliferation is still possible and, if so, sufficient to improve function. This lack of a clinically relevant chronic model also has implications for modRNA delivery. modRNA dosage has been tested previously, however only without MI [124] or in an acute post-MI setting [125]. To enhance modRNA delivery, lipid nanoparticles have been incorporated into the modRNA pipeline, but again testing has only occurred without MI [126] or in the acute environment [127]. These considerations are vital for cardiac modRNA therapeutic development as the use of clinically inferior models may impede clinical trial progression.

## Modified mRNA for genetic cardiac diseases

Although MI is the primary cause of HF [128], the condition has numerous aetiologies including coronary heart disease, cardiomyopathy, congenital heart disease and amyloidosis [129]. From numerous epidemiological studies [130–134], it is apparent that a major genetic component is intertwined in the development of these pathologies. For modelling and treating genetic diseases, the use of Cas9 has transformed medicine through genome editing which has transferred to the cardiovascular field (reviewed in [135]). However, only recently has modRNA been utilised to deliver the Cas9 mRNA with *in vivo* gene editing applied to treating transthyretin amyloidosis [48] and hereditary angioedema [136]. Whilst primarily known for its transient expression, modRNA has been utilised for protein replacement therapy of genetic disorders with clinical trials undertaken to treat methylmalonic acidemia [50], propionic acidaemia [51] and cystic fibrosis [52]. Although an emerging area, we highlight the published studies using modRNA for modelling and treating genetic conditions affecting CMs.

### Modelling genetic cardiac diseases

RNA binding motif protein 20 (RBM20) is a splicing factor that regulates alternative splicing of numerous cardiac genes encoding calcium-regulatory and sarcomeric proteins [137]. Autosomal dominant mutations of RBM20 comprise 2–6% of patients [138] with familial dilated cardiomyopathy (DCM). One specific mutation, R636S, results in protein mislocalisation and accumulation of cytoplasmic ribonucleoprotein (RNP) granules [139]. After demonstrating Rbm20^R636S^ iPSC-CMs exhibit cytoplasmic RNP granule formation, Nishiyama et al. [140] generated a Rbm20^R636S^ mouse model to test *in vivo* DCM therapies. Using base editing, Cas9 mRNA, Rbm20 sgRNA and ssODN were injected into mouse pronuclei and cytoplasm and transferred to a surrogate dam. Four-week-old mice exhibited decreased fractional shortening and elevated LV internal dimensions and by 12 weeks, atrial and ventricular dilation consistent with DCM. The work demonstrated how Cas9 mRNA can be applied for *in vivo* base editing providing an effective way to develop pathogenic mouse models.

Noonan syndrome (NS) is an autosomal dominant, multisystem disorder affecting around 1:1000 to 1:2500 live births [141]. NS is the second most common syndromic cause of congenital heart disease with multiple phenotypes including pulmonary stenosis, hypertrophic cardiomyopathy and/or atrial septal defect [142]. One NS-causative gene is the small GTPase, *RRAS2* [143], with overexpression of NS pathogenic variants in HEK293T cells resulting in impaired GTPase activity and hyperactivated ERK signalling [144]. To see whether NS RRAS2 mutants displayed similar effects in CMs, Batho and others transfected hPSC-CMs with RRAS2 WT, A70T and Q72L modRNA. After 4 hours, only RRAS2 mutants had significantly elevated phosphorylated ERK signalling, whilst after two days, CM size was increased indicating CM hypertrophy [145]. The research exemplifies how delivery of a mutated gene using modRNA can be used to model genetic diseases enabling high-throughput generation of disease models.

### Treating genetic cardiac diseases

Whilst modRNA has been used to treat hypercholesterolaemia through adenine base editing of *ANGPTL3* [146,147] and *PCSK9* [148–150] – the latter currently undergoing a phase 1b clinical trial [151] – the use of modRNA to directly treat CM genetic diseases is in its infancy with only a handful of published studies. Fabry disease is a rare X-linked disease caused by pathogenic mutations in the galactosidase alpha (*GLA*) gene [152]. GLA produces an enzyme that cleaves the terminal galactose of the glycosphingolipid, globotriaosylceramide (GB3) [152]. Fabry disease mutations result in GB3 buildup within lysosomes, resulting in clinical manifestations including chronic kidney disease and HF due to diastolic dysfunction [153]. After differentiating iPSC-CMs from Fabry disease patients showed GB3 accumulation and up-regulation of lysosomal-associated proteins, Ter Huurne and colleagues [154] assessed the therapeutic potential of a GLA modRNA. Overexpression of GLA modRNA in iPSC-CMs reduced GB3 levels and partially corrected expression of a subset of lysosomal proteins indicating rescue of the molecular phenotype and demonstrating the therapeutic potential of modRNA-mediated gene replacement therapy.

Duchenne muscular dystrophy (DMD), another X-linked disease, affects 1:3500 boys due to mutations in dystrophin (*DMD*) – a cytoskeletal protein required for myocyte membrane integrity. Lack of functional dystrophin causes muscle degeneration with death usually occurring by 25 through impaired breathing and/or cardiomyopathy [155]. To correct the genetic defect in a *mdx* DMD mouse model [156], Long et al. [157] delivered Cas9 mRNA, sgRNA and HDR template to mouse zygotes from *mdx* mice and implanted into pseudopregnant mice. Cas9-mediated editing resulted in a range of 2-100% gene correction of *DMD* with 41% editing sufficient to restore protein expression in the heart comparable with wildtype levels. Unlike skeletal muscle, no age-dependent dystrophin up-regulation occurred in the heart, likely due to reduced nucleation state and lack of local stem cells.

Hypertrophic cardiomyopathy (HCM) arises through mutations in a subset of sarcomere-associated genes with MYH7 and MYBPC3 responsible for ~50% of cases [158]. The manifestations are mutation-dependent; however, CM hypertrophy occurs, leading to thickened LV wall and often interstitial fibrosis and altered cardiac energetics [159]. To assess whether germline base editing could prevent disease onset in a *Myh6* R404Q (*MYH7* R403Q) HCM mouse model [160], Ma and colleagues [161] injected zygotes with a Cas9 base editor mRNA and sgRNA before surrogate implantation. After demonstrating safe and efficient editing in 84.4% of progeny, 25-week-old mice showed heart weight, fibrosis, CM area and LV wall thickness corrected to wildtype measurements. Furthermore, edited mice displayed corrected energy metabolic parameters indicated by basal oxygen consumption rate and metabolic gene expression comparable with wildtype levels.

Arrhythmogenic right ventricular cardiomyopathy (ARVC) is a disorder marked by progressive fibrofatty infiltration of the myocardium resulting in irregular rhythms [162]. Although ARVC exhibits genetic heterogeneity, most patients carry a mutation in genes that comprise the desmosome, including desmocollin-2 (*DSC2*) [163]. In a *Dsc2* knockout (KO) mouse model, the ARVC phenotype was recapitulated with hypertrophy and fibrosis resulting in systolic dysfunction and was shown to be mediated by Myl7 [164]. After showing that Dsc2 modRNA restored Myl7 protein expression in isolated CMs, adult *Dsc2* KO mice were given a single intracardiac injection which restored Dsc2 protein expression for three months. Restoration of Dsc2 was able to correct hypertrophy, fibrosis and ventricular dysfunction for up to three months. Encouragingly, these results were comparable with classical treatments of valsartan and fosinopril which require daily delivery [164].

Collectively, these studies illustrate the feasibility of modRNA as an approach for treating genetic cardiac diseases whilst demonstrating that Cas9 modRNA is an effective and safer gene editing tool. Whilst the Dsc2 modRNA data are encouraging, optimisation of the delivery strategy to enable intravenous delivery of the modRNA would advance the clinical application. Additionally, further investigation is required to determine whether successful gene editing can be achieved during neonatal or adult life to achieve beneficial outcomes for patients.

## Considerations for clinical translation

**Figure 3:**
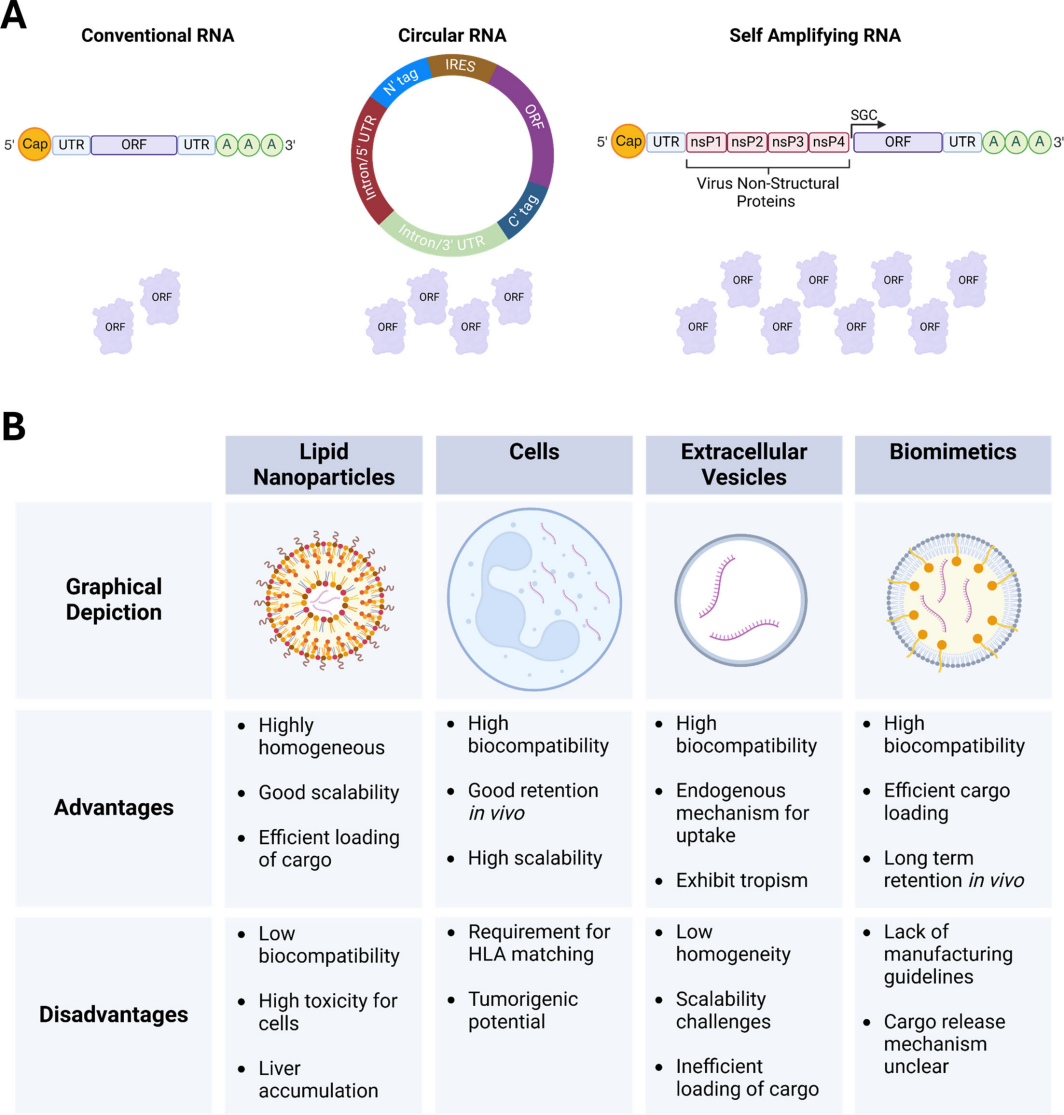
Alternative mRNA construct types and main packaging systems used for RNA therapeutic delivery. **(A**) Schematic showing the alternative mRNA construct types used for gene overexpression highlighting the structural differences and relative protein production between systems. Conventional linear modRNA is the primary type used with its transient nature well-suited for cardiac regeneration therapeutic delivery and safer gene editing. For enhanced protein expression, circular RNA (circRNA) are closed RNAs that use cap-independent translation to enable protein expression with engineering allowing development of cell-type specificities. The vector providing the largest level of protein expression across a greater duration is self-amplifying RNA (saRNA) which is engineered with non-structural proteins from viruses (often alphaviruses) to provide a replicating vector allowing long-term expression of therapeutics aligning it with treating chronic or genetic diseases. (**B**) The advantages and disadvantages of the main packaging systems used for modRNA delivery to cells. Whilst lipid nanoparticles have been the mainstay for RNA delivery for 60 years, they exhibit cellular toxicity resulting in the emergence of other delivery systems including the use of cells, extracellular vesicles and biomimetics – biological and synthetic hybrids. IRES, internal ribosome entry site; nsP, non-structural protein; SGC, subgenomic cap. Figure created with Biorender.com.

Hurdles are still limiting the clinical translation of modRNA; however, different avenues are being interrogated to improve the applicability of the modality. Whilst this review has focussed on conventional mRNA, the use of circular RNA (circRNA) and self-amplifying RNA (saRNA) is emerging in the RNA therapeutics field (Figure 3A). circRNA is a type of closed RNA lacking 5′ and 3′ ends that occurs naturally in cells and exhibits tissue-, development- and disease-specific expression [165]. As circRNAs lack a 5′ cap, most were classified as non-coding RNA until the discovery that circRNA use cap-independent translation for protein expression [166]. This has enabled synthetic circRNAs to be developed for therapeutic purposes to deliver non-coding [167,168] and protein-coding therapeutics with the latter currently used primarily in vaccine development [169]. Encouragingly, unmodified circRNA are also less immunogenic than unmodified conventional mRNA [170], increasing their appeal as a potential therapeutic vector. SaRNA utilises the non-structural proteins of single-stranded RNA viruses to produce a self-replicating vector for transgene expression [171]. Like conventional mRNA, saRNA is linear; however, due to its self-replication, it has been found that an influenza saRNA vaccine requires 64-fold less material to produce the same antigenic response [172]. Due to the presence of the viral proteins required for replication, saRNA exhibits enhanced immunogenicity, although this can be suppressed with the addition of anti-immunogenic genes [173]. However, inclusion of these genes increases an already large construct which can affect production. Together, circRNA and saRNA provide enhanced protein expression over longer durations, which reduces dosage whilst providing an overexpression vector better suited for treating chronic diseases. However, they have not yet been applied as a therapeutic modality in the cardiac medicine field.

Whilst great strides have been made to improve tissue targeting, more advanced *in vivo* delivery systems (Figure 3B) are required to enhance the translatability of modRNA. To prevent degradation by nucleases and enable cellular uptake and ultimately translation, most RNA therapeutics have relied on the use of lipid nanoparticles (LNPs) [174]. LNPs are highly homogeneous and scalable, but they exhibit low biocompatibility, high toxicity [175] and a strong propensity to accumulate in the liver [176], increasing the risk of side effects. Although unable to prevent liver accumulation, direct cardiac injection of LNP-encapsulated mRNA enables markedly reduced dosage [127,177] potentially reducing off-target effects. An alternative to LNPs is the use of cells as a delivery vector which sees the mRNA introduced to cells *ex vivo* before utilising the natural paracrine functions for mRNA delivery upon cellular injection [178]. This approach is far more biocompatible and extends longevity in circulation but is accompanied by limitations surrounding donor haplotype compatibility and potential tumourigenesis. Another biological approach is the use of extracellular vesicles – encompassing both exosomes and microvesicles – which are naturally produced thereby improving biocompatibility and utilising endogenous cellular uptake mechanisms (reviewed in [179]). Multiple examples exist where exosomes have been used for cardiac regeneration (reviewed in [180]) although they have not been used as a delivery vector to the heart. However, exosomes are highly heterogenous and of biological origin, limiting their scalability and cargo loading, which has been relatively inefficient, impeding their clinical translation. A newer approach has seen the development of biological and synthetic hybrids, known as biomimetics, which combine a synthetic core with a cellular membrane coating (reviewed in [181]). This combination enables efficient cargo loading into the core whilst the native cellular membrane reduces immunogenicity, providing high biocompatibility and increased stability [182]. However, the cargo release mechanism is currently unclear, and a lack of manufacturing criteria exists. Collectively, mRNA delivery system development is a focal point for its clinical translation, yet it is likely that the system used will be determined by the pathology and the need for transient or persistent mRNA translation.

**Figure 4:**
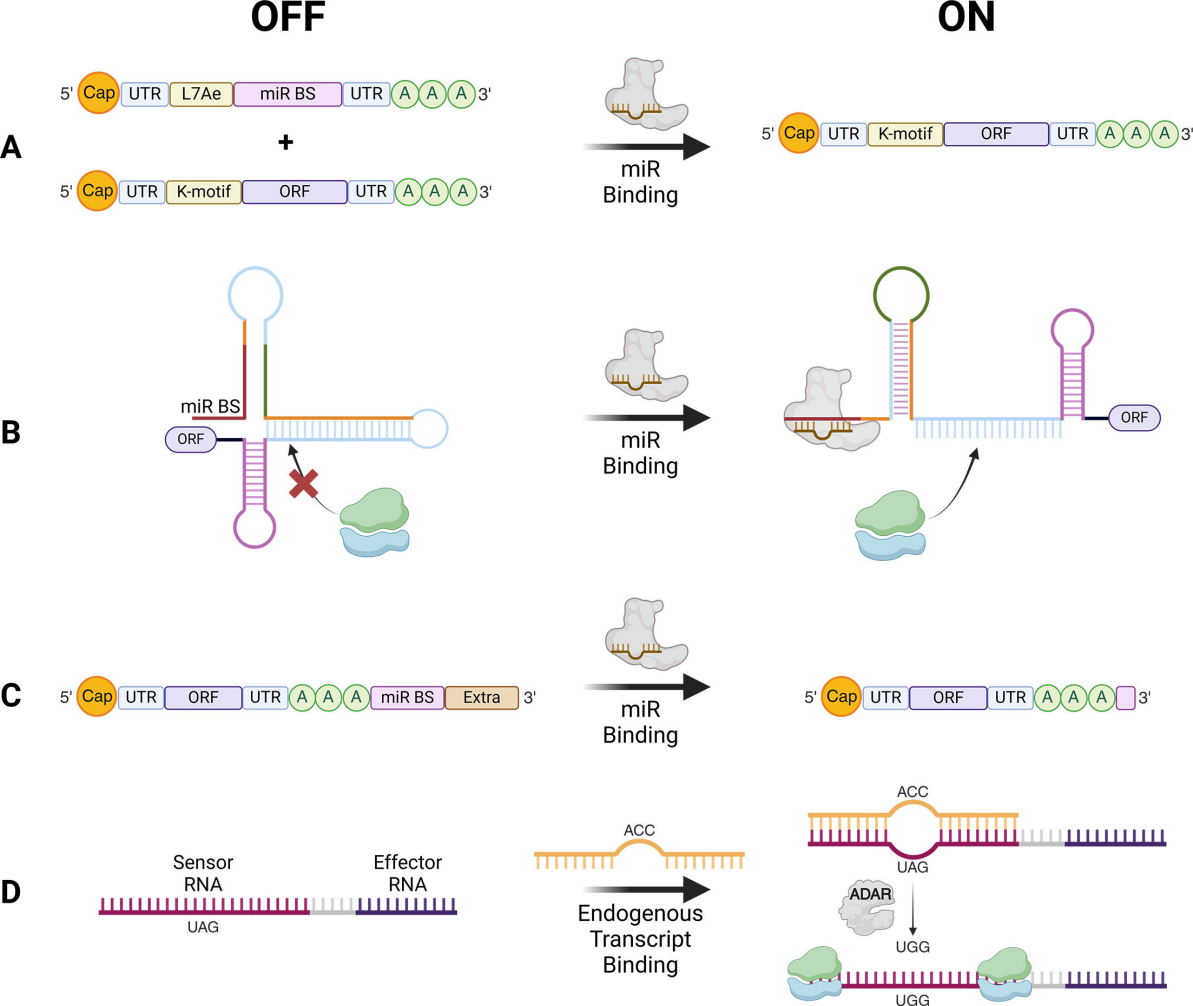
Sequence-dependent approaches to achieve cell type-specific translation. Schematic highlighting the emerging sequence-dependent tools being incorporated into modRNA design to achieve cell-specific translation. (**A**) L7Ae system utilises the archaeal ribosomal protein which upon binding to the K-motif, will induce degradation. In the presence of a cell-specific miRNA, this will instead bind and degrade the L7Ae-encoding modRNA enabling the modRNA encoding the ORF of interest to be translated. Other miRNA-dependent mechanisms are illustrated whereby miRNA binding will result in structural changes enabling ribosome entry (**B**) or revealing the poly(A) tail (**C**) allowing translation. (**D**) Harnessing the editing capabilities of ADAR, a sequence complementary to an endogenous transcript is incorporated upstream of the therapeutic. However, the complementary sequence contains a stop codon (UAG) meaning delivery to non-target cells will prevent translation. Conversely, uptake by target cells will edit the UAG to UGG allowing translation of the downstream product. miR BS, miRNA binding site. Figure created with Biorender.com.

Although packaging systems are also being developed to exhibit tropism [183], sequence-specific modRNA regulation is an evolving avenue for cell type-specific translation. This has been primarily employed in the cardiac regeneration field [104,107,114,116] through utilisation of a synthetic RNA circuit where the archaeal ribosomal protein, L7Ae, suppresses modRNA translation [184]. By incorporating a cell-specific miRNA binding site in the L7Ae-expressing modRNA, its degradation will occur in a cell-type specific manner, allowing translation of the therapeutic modRNA (Figure 4A). Alternative miRNA-dependent translational regulation of exogenous mRNA has been demonstrated with miRNA-binding site incorporation into the ribosome binding site [185] (Figure 4B) controlling mRNA translation; however, this was performed using circRNA. Another approach saw a miRNA-binding site incorporated in the poly(A) tail (Figure 4C) which upon miRNA binding, reveals the tail enabling translation and has been used to purify CMs from iPSC-CM differentiated culture [186]. Aside from miRNAs, there is a promising technology harnessing the double stranded RNA editing enzyme, ADAR, which edits mismatched adenosines to inosines – read as guanosine by the translational machinery [187]. Targeting cell-specific transcripts, modRNAs [188–191] are designed with a sequence complementary to the endogenous transcript except for an in-frame stop codon (UAG) which will be edited to tryptophan (UGG) enabling downstream translation in only the target cell (Figure 4D). Whilst in their early stages of development, these mechanisms are encouraging as they utilise cell state-specific transcript expression providing a versatile yet targeted approach to translational regulation.

## Summary and outlook

The use of modRNA for modelling and therapeutic intervention in cardiac pathologies is a constantly evolving and exciting space. The strengths of modRNA compared with other gene therapy vectors lie in its lack of gene packaging restrictions, reduced immunological response and transient but robust protein expression. The studies reviewed here demonstrated the importance of the last point for both pro-proliferative therapies and genome editing as the pulse-like expression enables safer outcomes by preventing prolonged CM de-differentiation and off-target effects, respectively. Whilst the future involvement of modRNA in cardiac research and medicine is anticipated, improved delivery systems are required to improve clinical translatability and the use of other mRNA systems is likely necessary to tackle chronic diseases. Addressing these challenges will allow modRNA to become a leading gene delivery vector for modelling and therapeutic purposes enabling the development of future treatments to combat HF.

**Perspectives**

- Modified mRNA (modRNA) is an emerging therapeutic modality due to its transient nature, low immunogenic response and flexible gene packaging capacity. These beneficial properties have seen it used for vaccine development and protein replacement therapy.
- The short-lasting translation of modRNA is well-suited for cardiac regeneration purposes where several studies have used modRNA to drive cardiomyocyte proliferation. More recently, Cas9 delivered using modRNA has resulted in effective editing enabling modelling and treatment of cardiac genetic diseases.
- Further improvements in modRNA delivery are necessary for its full clinical translation with a particular emphasis on packaging systems and cell type specificity. This development will enable further use of modRNA in both cardiac research and medicine.

## Abbreviations

AAVs: adeno-associated viruses
ARVC: arrhythmogenic right ventricular cardiomyopathy
CM: cardiomyocyte
DCM: dilated cardiomyopathy
DMD: Duchenne muscular dystrophy
GB3: globotriaosylceramide
HCM: hypertrophic cardiomyopathy
HF: heart failure
IVT: *in vitro* transcription
LAD: left anterior descending
LNPs: lipid nanoparticles
MI: myocardial infarction
NRCM: neonatal rat cardiomyocyte
ORF: open reading frame
RNP: ribonucleoprotein
TNF: tumour necrosis factor
UTRs: untranslated regions
circRNA: circular RNA
modRNA: modified mRNA
saRNA: self-amplifying RNA
